# Prognostic impact of human epidermal growth factor-like receptor 2 and hormone receptor status in inflammatory breast cancer (IBC): analysis of 2,014 IBC patient cases from the California Cancer Registry

**DOI:** 10.1186/bcr2225

**Published:** 2009-02-19

**Authors:** Jason A Zell, Walter Y Tsang, Thomas H Taylor, Rita S Mehta, Hoda Anton-Culver

**Affiliations:** 1 Department of Epidemiology, Genetic Epidemiology Research Institute, School of Medicine, University of California Irvine, 224 Irvine Hall, Irvine, CA 92697, USA; 2 Chao Family Comprehensive Cancer Center, University of California Irvine Medical Center, 101 The City Drive South, Orange, CA 92868, USA; 3 Division of Hematology/Oncology, Department of Medicine, School of Medicine, University of California Irvine Medical Center, 101 The City Drive South, Orange, CA 92868, USA; 4 Department of Medicine, School of Medicine, University of California Irvine Medical Center, 101 The City Drive South, Orange, CA 92868, USA

## Abstract

**Introduction:**

Inflammatory breast cancer (IBC) is an aggressive form of breast cancer associated with overexpression of Her2/Neu (human epidermal growth factor-like receptor 2 (HER2)) and poor survival. We investigated survival differences for IBC patient cases based on hormone receptor status and HER2 receptor status using data from the California Cancer Registry, as contrasted with locally advanced breast cancer (LABC), metastatic breast cancer (MBC) and non-T4 breast cancer.

**Methods:**

A case-only analysis of 80,099 incident female breast cancer patient cases in the California Cancer Registry during 1999 to 2003 was performed, with follow-up through March 2007. Overall survival (OS) and breast cancer-specific survival (BC-SS) were analyzed using Kaplan–Meier methods and Cox proportional hazards ratios.

**Results:**

A total of 2,014 IBC, 1,268 LABC, 3,059 MBC, and 73,758 non-T4 breast cancer patient cases were identified. HER2^+ ^was associated with advanced tumor stage (*P *< 0.0001). IBC patient cases were more likely to be HER2^+ ^(40%) and less likely to be hormone receptor-positive (HmR^+^) (59%) compared with LABC (35% and 69%, respectively), MBC (35% and 74%), and non-T4 patient cases (22% and 82%). HmR^+ ^status was associated with improved OS and BC-SS for each breast cancer subtype after adjustment for clinically relevant factors. In multivariate analysis, HER2^+ ^(versus HER2^-^) status was associated with poor BC-SS for non-T4 patient cases (hazards ratio = 1.16, 95% confidence interval 1.05 to 1.28) and had a borderline significant association with improved BC-SS for IBC (hazards ratio = 0.82, 95% confidence interval = 0.68 to 0.99).

**Conclusions:**

Despite an association with advanced tumor stage, HER2^+ ^status is not an independent adverse prognostic factor for survival among IBC patient cases.

## Introduction

Inflammatory breast cancer (IBC) is an aggressive disease characterized biologically by dermal lymphatic invasion with tumor emboli and angiogenesis, and characterized clinically by breast tenderness and skin induration overlying the breast, typically without underlying palpable mass [1]. IBC is a rapidly progressive tumor, with propensity for metastatic tumor spread, and poor overall survival (OS) compared with non-IBC locally advanced breast cancer (LABC). Although relatively rare (that is, 2% of all breast cancer patient cases), time-trend data from the US National Cancer Institute Surveillance, Epidemiology, and End Results (SEER) database suggest that the incidence of IBC may be increasing [2,3].

Despite multidisciplinary treatment with chemotherapy, radiation and surgery, IBC still carries a poor prognosis, with a 5-year survival rate of about 30% [4], with no significant change in the prognosis over the past 30 years [5]. Advances in the treatment of IBC have been hampered by a general lack of prognostic or predictive parameters, in part because the rarity of this tumor has made it difficult to conduct large clinical trials [6]. Population-based analyses of SEER data reveal that IBC has distinct epidemiologic differences from LABC and non-T4 breast cancer [2]. For example, IBC patient cases with estrogen receptor (ER)-negative tumors were shown to have decreased breast cancer-specific survival (BC-SS) compared with those with ER-positive tumors (2.0 years vs. 4.0 years). In addition, several small studies have shown that a high proportion of IBC tumors are positive for Her2/Neu (human epidermal growth factor-like receptor 2 (HER2)) receptor compared with historic data for non-T4 patient cases [7-9]. HER2 is a proto-oncogene located on chromosome 17. It is overexpressed in about 25% to 30% of breast cancer and in general is associated with a more aggressive breast cancer phenotype [10]. There has been interest in investigating the role of HER2 receptor status in IBC survival, but recording of HER2 receptor status is not available in the SEER database. Previously Caggiano and colleagues used data from the California Cancer Registry (CCR) to show that HER2 status did not differentiate survival among ER-negative/progesterone receptor (PR)-negative patient cases [11]. However, results for IBC were not reported. One report indicates that HER2 receptor status is not an independent risk factor for survival in ER-negative/PR-negative invasive breast cancer in general [12]. Another report shows that HER2 status does not significantly affect recurrence-free survival in both univariate and multivariate models among 179 IBC patients [13]. Thus far, however, the usefulness of HER2 receptor status as a prognostic factor for survival among IBC patient cases in a population-based setting is still unclear.

In the present study, we use data from the CCR to estimate differences in OS for IBC, LABC, metastatic breast cancer (MBC) and non-T4 breast cancer based on hormone and HER2 receptor status. In doing so, our goal is to evaluate these potential prognostic factors for survival after diagnosis of IBC. We have restricted our analyses to a period prior to 2005, when trastuzumab became widely used in the adjuvant setting for treatment of locoregional breast cancer [14,15].

## Materials and methods

### Study population

We performed a retrospective, case-only analysis of 80,099 incident female breast cancer patient cases in CCR during 1999 to 2003 with follow-up through March 2007. The CCR is part of the National Cancer Institute's SEER program, and is the largest contiguous-area, population-based cancer registry in the world [16]; standardized data collection and quality control procedures have been in place since 1988 [17-19]. Data were abstracted from medical and laboratory records by trained tumor registrars. The tumor site and histology were coded according to World Health Organization criteria in the International Classification of Diseases for Oncology [20]. Patient cases were extracted based on the SEER primary site recode 26000 for breast cancer. IBC, LABC, and non-T4 breast cancer were identified using the extent of disease (EOD) coding for tumor extension and tumor size in addition to pathologic International Classification of Diseases ICD-O-3 coding, as has been done previously [2].

The SEER extent of disease and surgical staging variables were used to derive tumor node metastasis (TNM) data. Patient cases identified through death certificate or autopsy only were excluded. Recorded data included clinical information such as stage at presentation, histology, treatment during the first course of therapy, and vital status. Socioeconomic status (SES) is recorded as a single index variable in the CCR using statewide measures of education, income, and occupation from census data, as described previously [21]. The SES variable used is described in full elsewhere [22-24]. Quintiles for the SES score were analyzed. Cause of death was recorded according to International Classification of Diseases criteria in effect at the time of death. The last date of follow-up was either the date of death or the last date of contact.

### Hormone-receptor and Her2/Neu status

We classified patient cases as hormone receptor-positive if either the ER marker or the PR marker was positive, and as hormone receptor-negative if both markers were negative. Otherwise, patient cases were classified as hormone receptor unknown. Neither assay type nor titer was available in the dataset for either of these markers. HER2 status was classified as positive or negative based on the coding in the CCR. The method of detection for HER2 status (that is, fluorescent *in situ *hybridization, immunohistochemistry) was not available.

### Statistical analysis

Associations between categorical and dichotomous variables were tested using Pearson's chi-squared test or Fisher's exact test. Differences in continuous variables across more than two groups were tested with the nonparametric Kruskall–Wallis test. Survival estimates were generated with Kaplan–Meier methods and compared with the log-rank test. Cox proportional-hazards methods were used to compare OS and BC-SS while adjusting for covariates. Covariates included in the multivariate regression model included those factors known to have prognostic value in breast cancer, including age (years), ethnicity, tumor grade, SES quintile, treatment with surgery, radiation therapy, and chemotherapy. All statistical analyses were conducted using SAS 9.1 statistical software (SAS Institute Inc., Cary, NC, USA). Statistical significance required a two-tailed *P *value < 0.05.

### Ethical considerations

The present study involved analysis of extant CCR data with no subject intervention. No identities were linked to subject records. This study was approved by the University of California Irvine Institutional Review Board under the category of exempt status, and no consent form was required from the participants (IRB#2006-5264).

## Results

### Demographic comparisons

Among the 80,099 incident patient cases identified in this analysis were 2,014 IBC, 1,268 LABC, 3,059 MBC, and 73,758 non-T4 patient cases. Comparison of relevant clinicopathologic variables across the four major breast cancer categories analyzed is presented in Table 1. With a median age at diagnosis of 57.3 years, IBC patient cases were younger in age than LABC, MBC or non-T4 breast cancer patient cases. A greater proportion of African Americans and Hispanics were observed among IBC and LABC cases compared with non-T4 patient cases. A full 99% of non-T4 patient cases received surgical treatment, compared with 75% for IBC cases, 83% for LABC cases, and 44% for MBC cases. Treatment with radiation therapy involved 49% of non-T4 and IBC patient cases, 39% for LABC patient cases, and 33% for MBC patient cases. A greater proportion of IBC patient cases received treatment with chemotherapy compared with LABC, MBC or non-T4 patient cases. Non-T4 breast cancer patient cases were more likely to be in the highest two SES quintiles than were IBC, LABC, or MBC patient cases.

**Table 1:** Demographic characteristics for invasive breast cancer cases by each of the four investigated categories^a^

	Inflammatory breast cancer (n = 2,014)	Locally advanced breast cancer (n = 1,268)	Metastatic breast cancer (n = 3,059)	Non-T4 breast cancer (n = 73,758)	Total (n = 80,099)
Median (± SD) age at diagnosis (years)	57.3 ± 14.4	64.3 ± 16.3	61.9 ± 14.9	60.2 ± 13.8	60.2 ± 13.9
Race					
Caucasian	1,252 (62%)	783 (62%)	2,009 (66%)	52,280 (71%)	56,324 (70%)
African-American	192 (10%)	126 (10%)	287 (9%)	4,008 (5%)	4,613 (6%)
Hispanic	412 (20%)	220 (17%)	503 (16%)	10,188 (14%)	11,323 (14%)
Asian	140 (7%)	122 (10%)	241 (8%)	6,808 (9%)	7,311 (9%)
Other	18 (0.9%)	17 (1%)	19 (0.6%)	474 (0.6%)	528 (0.7%)
Stage					
I	-	-	-	38,571 (52%)	38,571 (48%)
IIA	-	-	-	21,308 (29%)	21,308 (27%)
IIB	-	-	-	11,137 (15%)	11,137 (14%)
IIIA	-	-	-	2,675 (4%)	2,675 (3%)
IIIB	1,520 (75%)	1,268 (100%)	-	67 (<0.1%)	2,855 (4%)
IV	494 (25%)	0 (0%)	3,059 (100%)	0 (0%)	3,553 (4%)
Tumor grade					
Grade 1	51 (3%)	82 (6%)	141 (5%)	16,458 (22%)	16,732 (21%)
Grade 2	433 (22%)	363 (29%)	764 (25%)	28,873 (39%)	30,433 (38%)
Grade 3	1,025 (51%)	616 (49%)	1,108 (36%)	21,466 (29%)	24,215 (38%)
Grade 4	95 (5%)	38 (3%)	81 (3%)	1,536 (2%)	1,750 (2%)
Unknown	410 (20%)	169 (13%)	965 (32%)	5,425 (7%)	6,969 (9%)
Estrogen receptor					
Estrogen receptor-positive	862 (56%)	653 (67%)	1,533 (73%)	49,960 (80%)	53,008 (79%)
Estrogen receptor-negative	675 (44%)	319 (33%)	573 (27%)	12,419 (20%)	13,986 (21%)
Missing	477	296	953	11,379	13,105
Progesterone receptor					
Progesterone receptor-positive	666 (45%)	511 (54%)	1,123 (58%)	40,555 (68%)	42,855 (67%)
Progesterone receptor-negative	819 (55%)	430 (46%)	826 (42%)	19,137 (32%)	21,212 (33%)
Missing	529	327	1,110	14,066	16,032
Hormone receptor^b^					
Hormone receptor-positive	916 (60%)	671 (69%)	1,578 (75%)	51,062 (82%)	54,227 (81%)
Hormone receptor-negative	626 (40%)	303 (31%)	533 (25%)	11,499 (18%)	12,961 (19%)
Missing	472	294	948	11,197	12,911
HER2					
HER2-positive	477 (40%)	255 (35%)	506 (35%)	9,575 (22%)	10,813 (23%)
HER2-negative	703 (60%)	484 (65%)	942 (65%)	34,754 (78%)	36,883 (77%)
Missing	834	529	1,611	29,429	32,403
Surgical treatment					
None	511 (25%)	218 (17%)	1,707 (56%)	612 (<1%)	3,048 (4%)
Mastectomy/lumpectomy/other	1,500 (75%)	1,050 (83%)	1,352 (44%)	73,145 (99%)	77,047 (96%)
Unknown	3 (0.2%)	0 (0%)	0 (0%)	1 (0%)	4 (0%)
Radiation therapy					
None	1,022 (51%)	779 (61%)	2,041 (67%)	37,334 (51%)	41,176 (51%)
Any	992 (49%)	489 (39%)	1,018 (33%)	36,422 (49%)	38,921 (49%)
Unknown	0 (0%)	0 (0%)	0 (0%)	2 (0%)	2 (0%)
Chemotherapy					
None	351 (17%)	537 (42%)	1,345 (44%)	44,474 (60%)	46,707 (58%)
Any	1,625 (81%)	683 (54%)	1,606 (53%)	27,428 (37%)	31,342 (39%)
Unknown	38 (2%)	48 (4%)	108 (4%)	1,856 (3%)	2,050 (3%)
Socioeconomic status					
Lowest	358 (18%)	235 (19%)	509 (17%)	7,786 (11%)	8,888 (11%)
Second lowest	379 (19%)	263 (21%)	584 (19%)	11,900 (16%)	13,126 (16%)
Middle	427 (21%)	265 (21%)	631 (21%)	15,376 (21%)	16,699 (21%)
High	465 (23%)	252 (20%)	705 (23%)	17,608 (24%)	19,030 (24%)
Highest	385 (19%)	253 (20%)	630 (21%)	21,088 (29%)	22,356 (28%)

^a^California Cancer Registry data, 1999 to 2004. *P *< 0.0001 for comparisons of each variable across the four major breast cancer subtypes. HER2, human epidermal growth factor-like receptor 2; SD, standard deviation. ^b^Hormone receptor-positive indicates estrogen receptor-positive and/or progesterone receptor-positive; hormone receptor-negative indicates estrogen receptor-negative and progesterone receptor-negative.

### Breast tumor receptor status

Hormone receptor status was available for 84% of the study cohort. Table 2 reveals the hormone receptor status by stage at presentation for each of the investigated breast cancer categories. ER-positive and PR-positive status was highest among non-T4 patient cases (80% ER, 68% PR), particularly those with early-stage disease. Expression of ER and PR, respectively, was lower among IBC patient cases (56%, 45%), LABC patient cases (67%, 54%) and MBC patient cases (73%, 58%). A greater proportion of stage IV IBC patient cases had ER-positive tumors compared with stage IIIB patient cases (60% vs. 55%), but equal proportions had PR-positive tumors (45% vs. 45%).

**Table 2:** Hormone receptor and HER2 receptor status by stage at presentation for investigated breast cancer categories^a^

	Inflammatory breast cancer		Locally advanced breast cancer	Metastatic breast cancer	Non-T4 breast cancer				
	
	Stage IIIB	Stage IV	Stage IIIB	Stage IV	Stage I	Stage IIA	Stage IIB	Stage IIIA	Stage IIIB
Estrogen receptor status									
Estrogen receptor-negative	536 (45%)	139 (40%)	319 (33%)	573 (27%)	4,836 (15%)	4,323 (24%)	2,510 (26%)	730 (32%)	20 (36%)
Estrogen receptor-positive	655 (55%)	207 (60%)	653 (67%)	1,533 (73%)	27,491 (85%)	13,778 (76%)	7,091 (74%)	1,565 (68%)	35 (64%)
Number missing	329	148	296	953	6244	3,207	1,536	380	12
Progesterone receptor status									
Progesterone receptor-negative	640 (55%)	179 (55%)	430 (46%)	826 (42%)	8,482 (28%)	6,166 (35%)	3,495 (38%)	973 (44%)	21 (41%)
Progesterone receptor-positive	517 (45%)	149 (45%)	511 (54%)	1,123 (58%)	22,326 (72%)	11,222 (65%)	5,748 (62%)	1,229 (56%)	30 (59%)
Number missing	363	166	327	1,110	7,763	3,920	1,894	473	16
Hormone receptor status									
Hormone receptor-negative	502 (42%)	124 (36%)	303 (31%)	533 (25%)	4,426 (14%)	4,021 (22%)	2,351 (24%)	683 (30%)	18 (33%)
Hormone receptor-positive	694 (58%)	222 (53%)	671 (69%)	1,578 (75%)	28,003 (86%)	14,138 (78%)	7,267 (76%)	1,617 (70%)	37 (67%)
Number missing	324	148	294	948	6,142	3,149	1,519	375	12
HER2 receptor status									
HER2-negative	556 (60%)	147 (57%)	484 (65%)	942 (65%)	18,280 (82%)	10,110 (77%)	5,104 (72%)	1,235 (70%)	25 (64%)
HER2-positive	367 (40%)	110 (43%)	255 (35%)	506 (35%)	4,058 (18%)	2,953 (23%)	2,010 (28%)	540 (30%)	14 (36%)
Number missing	597	237	529	1,611	16,233	8,245	4,023	900	28

^a^California Cancer Registry data, 1999 to 2004. HER2, human epidermal growth factor-like receptor 2.

HER2 receptor status was available for 60% of the breast cancer patient cases in this study. Among those with data available for HER2, IBC patient cases were noted to have a higher proportion of HER2-positive patient cases (stage IIIB 40%, stage IV 43%) compared with non-T4 patient cases (stage I, 18%; stage IIA, 23%; stage IIB, 28%; stage IIIA, 30%; stage IIIB, 36%), LABC patient cases (35%) or MBC patient cases (35%).

### Cause of death and survival analysis by breast cancer subtype

Overall there were 13,991 deaths among the 80,099 patient cases analyzed (17.5%). Cause of death was recorded as follows: breast cancer (5,963), heart disease (1,340), other causes (2,593), and missing (4,109). Among those suffering death in this study, breast cancer was responsible for 72% of IBC deaths, 55% of LABC deaths, 73% of MBC deaths, and 31% of non-T4 deaths (*P *< 0.0001).

**Figure 1:**
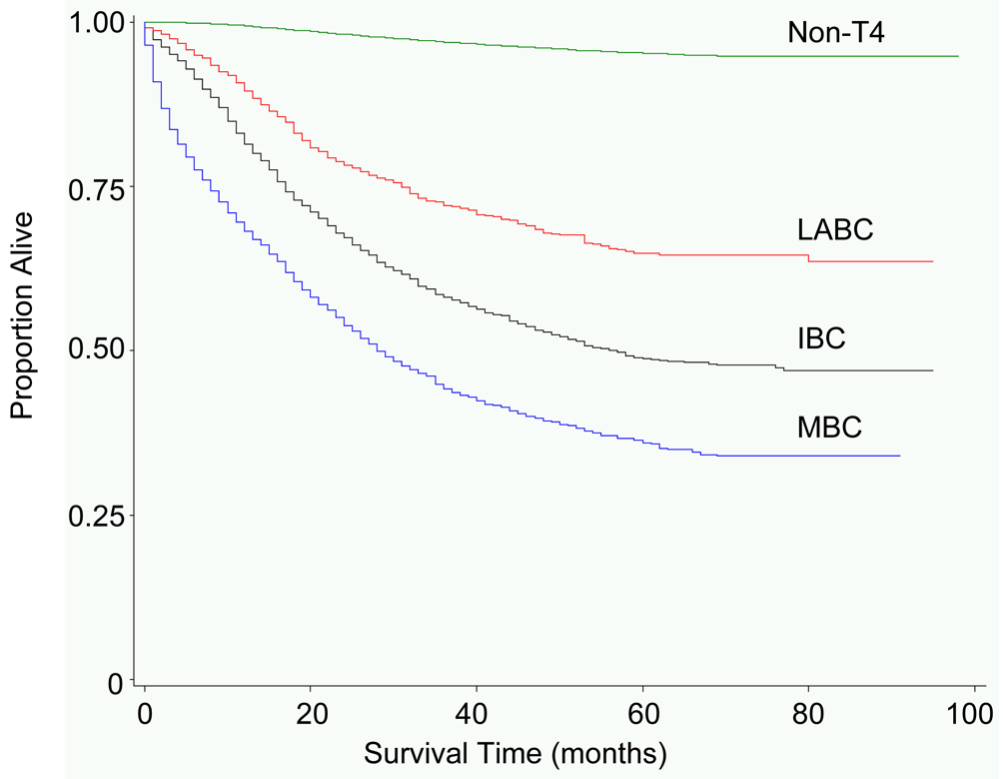
Breast cancer-specific survival by breast cancer subtype. Survival in breast cancer patient cases diagnosed during 1999 to 2003 with follow-up through March 2007, from the California Cancer Registry. IBC = inflammatory breast cancer; LABC = locally advanced breast cancer; MBC = metastatic breast cancer; non-T4, non-T4 breast cancer.

Univariate BC-SS analysis revealed significant differences in survival across the major breast cancer categories (Figure 1). Non-T4 patient cases (5-year BC-SS = 95%; median BC-SS not reached (NR) at >95 months) had significantly improved BC-SS compared with LABC patient cases (5-year BC-SS = 65%; median BC-SS NR at >95 months), IBC patient cases (5-year BC-SS = 49%; median BC-SS = 57 months, 95% confidence interval (CI) = 50 to 77), or MBC patient cases (5-year BC-SS = 36%; BC-SS = 28 months, 95% CI = 27 to 31) (*P *< 0.0001).

### Survival analysis by breast tumor receptor status

Univariate and multivariate survival analyses using Cox proportional hazards models for each of the four major breast cancer subtypes by breast tumor receptor status are presented in Tables 3 and 4. Hormone receptor-positive status (compared with hormone receptor-negative status as a referent) was associated with improved OS and BC-SS for IBC, LABC, MBC, and non-T4 patient cases in univariate survival analysis, and also after adjustment for age, race, grade, SES, surgery, radiation therapy, and chemotherapy (Table 3). HER2^+ ^status, however, was not associated with OS for IBC, LABC, or MBC patient cases in adjusted or unadjusted analyses (Table 4).

**Table 3:** Overall survival and breast cancer-specific survival for breast cancer cases by tumor hormone receptor status^a^

	Inflammatory breast cancer		Locally advanced breast cancer		Metastatic breast cancer		Non-T4 breast cancer	
	
	Positive^b^	Not positive^b^	Positive	Not positive	Positive	Not positive	Positive	Not positive
Overall mortality								
Number of events	427	494	185	306	432	1,069	2,330	5,789
Number at risk	626	916	303	671	533	1,578	11,499	51,062
Unadjusted HR (95% CI)	1 (ref)	0.62 (0.55 to 0.71)	1 (ref)	0.56 (0.47 to 0.68)	1 (ref)	0.60 (0.53 to 0.67)	1 (ref)	0.52 (0.49 to 0.54)
Adjusted HR (95% CI)^c^	1 (ref)	0.64 (0.56 to 0.73)	1 (ref)	0.49 (0.40 to 0.60)	1 (ref)	0.54 (0.48 to 0.61)	1 (ref)	0.61 (0.58 to 0.64)
Breast cancer-specific mortality								
Number of events	338	318	138	129	340	739	1,239	1,313
Number at risk	626	916	303	671	533	1,578	11,499	51,062
Unadjusted HR (95% CI)	1 (ref)	0.52 (0.45 to 0.61)	1 (ref)	0.33 (0.26 to 0.42)	1 (ref)	0.54 (0.48 to 1.62)	1 (ref)	0.22 (0.21 to 0.24)
Adjusted HR (95% CI)^c^	1 (ref)	0.55 (0.47 to 0.65)	1 (ref)	0.33 (0.25 to 0.43)	1 (ref)	0.51 (0.44 to 0.58)	1 (ref)	0.43 (0.39 to 0.47)

^a^Univariate and multivariate adjusted analysis of overall survival and breast cancer-specific survival. Data for cases with missing hormone receptor status suppressed from table. ^b^Data presented as number of cases: not positive, not hormone receptor-positive; positive, hormone receptor-positive; ref, reference. ^c^Includes adjustment for age (years), ethnicity, grade, socioeconomic status quintile, treatment with surgery, radiation therapy, and chemotherapy. HR, hazards ratio; CI, confidence interval.

**Table 4:** Overall survival and breast cancer-specific survival for breast cancer cases by tumor HER2 receptor status^a^

	Inflammatory breast cancer		Locally advanced breast cancer		Metastatic breast cancer		Non-T4 breast cancer	
	
	Positive^b^	Not positive^b^	Positive	Not positive	Positive	Not positive	Positive	Not positive
Overall mortality								
Number of events	400	280	239	131	648	347	4,128	1,397
Number at risk	703	477	484	255	942	506	34,754	9,575
Unadjusted HR (95% CI)	1 (ref)	0.99 (0.85 to 1.15)	1 (ref)	1.01 (0.82 to 1.26)	1 (ref)	0.99 (0.87 to 1.12)	1 (ref)	1.19 (1.12 to 1.27)
Adjusted HR (95% CI)^c^	1 (ref)	0.87 (0.75 to 1.02)	1 (ref)	1.00 (0.81 to 1.24)	1 (ref)	0.88 (0.77 to 1.01)	1 (ref)	1.05 (0.99 to 1.12)
Breast cancer-specific mortality								
Number of events	282	193	132	78	450	266	1,185	556
Number at risk	703	477	484	255	942	506	34,754	9,575
Unadjusted HR (95% CI)	1 (ref)	0.98 (0.81 to 1.17)	1 (ref)	1.11 (0.84 to 1.47)	1 (ref)	1.09 (0.94 to 1.27)	1 (ref)	1.68 (1.52 to 1.86)
Adjusted HR (95% CI)^c^	1 (ref)	0.82 (0.68 to 0.99)	1 (ref)	0.96 (0.72 to 1.28)	1 (ref)	0.95 (0.82 to 1.11)	1 (ref)	1.16 (1.05 to 1.28)

^a^Univariate and multivariate adjusted analysis of overall survival and breast cancer-specific survival. Data for cases with missing hormone receptor status suppressed from table. ^b^Data presented as number of cases: not positive, not human epidermal growth factor-like receptor 2 (HER2)-positive; positive, HER2-positive; ref, reference. ^c^Includes adjustment for age (years), ethnicity, grade, socioeconomic status quintile, treatment with surgery, radiation therapy, and chemotherapy. HR, hazards ratio; CI, confidence interval.

Non-T4 breast cancer patient cases were observed to have an association with HER2^+ ^status and poor OS on univariate analysis (hazards ratio = 1.19 vs. HER2^-^, 95% CI = 1.12 to 1.27), which was borderline nonsignificant on multivariate analysis. In multivariate analysis, HER2^+ ^(versus HER2^-^) status was associated with poor BC-SS for non-T4 patient cases (hazards ratio = 1.16, 95% CI = 1.05 to 1.28), had no association with BC-SS for LABC patient cases (hazards ratio = 0.96, 95% CI = 0.72 to 1.28) or MBC patient cases (hazards ratio = 0.95, 95% CI = 0.82 to 1.11), and had a borderline significant association with improved BC-SS for IBC patient cases (hazards ratio = 0.82, 95% CI = 0.68 to 0.99) after adjustment for age, race, grade, SES, surgery, radiation therapy, and chemotherapy (Table 4).

## Discussion

As expected, we observed that BC-SS is lower for IBC patient cases compared with non-T4 and LABC patient cases (Figure 1). Similar to what we observed for LABC, MBC, and non-T4 breast cancer patient cases, hormone receptor-positive status was associated with improved BC-SS and OS among IBC patient cases, even after adjustment for HER2 status and other clinically relevant factors (Table 3). These findings are generally in agreement with the prior study by Brown and colleagues showing that, in invasive breast cancer, hormone receptor-negative status rather than HER2 status is a major contributor to poor survival [12]. We observed that the proportion of HER2-positive tumors is greater among IBC patient cases (40% in stage IIIB and 43% in stage IV) than LABC, MBC, or non-T4 patient cases. These findings are consistent with previous studies worldwide estimating HER2 positivity at 36% to 50% of IBC patients [9,25]. This conserved rate of HER2 positivity among IBC patient cases occurs even in areas of the world with historically higher incidence of the disease, such as Tunisia – where IBC accounts for approximately 6% of all breast cancer (contrasted with 2.5% IBC in our study) [26,27].

Despite reported observations that HER2 positivity occurs more frequently in IBC, very few studies have specifically examined whether HER2 receptor status was prognostic for survival in IBC patients. Dawood and colleagues reported that, in the absence of treatment with the HER2 receptor antibody trastuzumab, there is no statistically significant difference in recurrence-free survival observed between IBC patients with HER2-positive breast cancer compared with HER2-negative breast cancer [13]. Interestingly, after adjusting for patient and tumor characteristics, patients with HER2-positive disease had increased OS, presumably because most of them received trastuzumab for their recurrent disease. Trastuzumab may therefore play an important role in treatment of metastatic or recurrent IBC. Sawaki and colleagues also did not find HER2 positivity to be a significant prognostic factor in IBC; however, their study was probably underpowered to detect such a difference due to the small sample size (that is, an analysis of 46 patients within a single Japanese hospital) [9]. We obtained similar results in the present study, involving a large population-based database and sufficient statistical power to detect small differences. We observed no statistically different BC-SS characteristics for IBC patient cases based on HER2 receptor status at the time of diagnosis on unadjusted analysis, and a borderline significantly improved survival for patient cases with HER2^+ ^tumors in the BC-SS adjusted analysis. Additionally, in the present analysis of data from the largest geographically contiguous cancer registry in the world, we found that HER2 is not prognostic for overall survival among LABC or MBC patient cases, and that HER2-positive status is associated with poor overall survival and BC-SS only among non-T4 breast cancer patient cases.

There are several possible explanations for why HER2 is not prognostic of decreased survival in IBC. IBC's aggressive disease characteristics may be unrelated to the presence of HER2 receptor or the HER2 receptor tyrosine kinase activity. Various theories have been proposed. For example, IBC tumors are known to be highly angiogenic and angioinvasive, with expression of proangiogenic factors that may contribute to early metastatic disease [28,29], which therefore could be related to poor survival. Increased expression of chemokine receptors such as CCR7 [30] and genes that are related to higher metabolic rates such as Ki-67 [31] may also contribute adversely to survival. Numerous other molecules may contribute to IBC's aggressive behavior, including E-cadherin [32], Rho proteins, and WISP3 [33,34]. Some of these molecules may be responsible for micrometastatic disease early on and thus increase IBC's tendency for recurrence [35]. Future studies may determine whether expression of these molecules is prognostic of poor survival in IBC. Finally, it has been proposed that an anthracycline-based chemotherapy regimen may be more effective in HER2-positive tumors compared with HER2-negative ones because HER2-positive tumors often have higher expression of topoisomerase II, which is the target for anthracyclines [13]. Selective killing of HER2 positive tumor cells with anthracycline-based chemotherapy (with or without anti-HER2 trastuzumab treatment) could therefore possibly eliminate HER2 positivity's impact on recurrence-free survival and OS.

Since HER2 receptor status is not prognostic for decreased survival in IBC patients, it calls into question whether antibodies directed to the HER2 receptor, such as Herceptin, may improve survival in these patients. We were not able to perform this analysis in our study because data on specific chemotherapeutic agents utilized are not available in the CCR. As described previously, in one relatively small study, trastuzumab appeared to have improved survival in recurrent or metastatic IBC, and HER2 positivity was associated with a trend towards higher overall survival [13]. A strength of the study design is that we restricted our analyses to the period prior to 2005, when trastuzumab became widely used in the adjuvant setting for treatment of locoregional breast cancer [14,15]; our analyses should therefore not be confounded by this landmark event.

Currently there are no specific treatment guidelines for IBC. These IBC patients are often treated the same way as LABC patient cases, with intensive adjuvant or neoadjuvant chemotherapy in addition to surgery and radiation therapy. Further trials involving larger numbers of subjects with adequate power are warranted to assess the efficacy of trastuzumab in HER2-positive IBC. It is interesting that HER2 receptor status is not an adverse prognostic factor for survival after diagnosis of IBC, and yet trastuzumab may still benefit IBC patients, a finding that warrants further research. It is noteworthy that the BC-SS estimates for IBC noted in the CCR during the period 1999 to 2003 are higher than previous estimates in the SEER during the period 1988 to 2000. Many factors could explain this difference, as management strategies have evolved over time. Optimal administration schedules of anthracyclines and taxanes have been adopted in routine practice. For example, dose-dense anthracycline-based chemotherapy [36,37], dose intensification therapy [38], and metronomic (weekly) taxane regimens [37,39,40] are associated with improved outcomes in breast cancer. More recently, use of trastuzumab at time of diagnosis has shown increased pathologic complete response in HER2-positive IBC, and promising progression-free survival has been reported with early follow-up [41,42]. In as much as pathologic complete response is a surrogate of prolonged survival in IBC, future population-based data after 2005 may reveal further HER2-positive survival improvements among individuals with IBC.

There are several limitations to the present study. First, a large proportion of the data were missing for ER (16%), PR (20%), and HER2 (31%) tumor receptor status. All multivariate models were therefore analyzed with inclusion of patient cases lacking tumor receptor status. The level of HER2 amplification was not recorded in the cancer registry database. Additionally, there are no available data on the specific chemotherapy agents that patients received. It is unclear how many people received trastuzumab, for example, or received other therapy that might have affected survival. Only 81% of IBC patient cases received chemotherapy, only 75% received surgery, and 49% received radiotherapy. While these rates are low compared with what one might expect from clinical trial data, population-based data typically have lower proportions of cases receiving treatment, as all patients are included in the analysis (that is, even those with severe comorbid conditions and poor performance status). Consistent with other population-based research, there is also no information in the CCR reporting performance status or comorbid conditions that might influence survival. Finally, these population-based data came from a wide variety of sources, and thus variation of reference values for tests of hormone receptor status probably exists.

## Conclusions

Our observations help characterize the relationship between IBC, HER2 receptor status and the effects of HER2 status effects on survival. Based on these observations, we conclude that IBC's poor survival is probably unrelated to HER2 status. Rather, the aggressive biological nature of IBC or other clinical factors may explain the poor OS for IBC. Optimization of chemotherapy schedules may have preferentially improved the outcome of HER2-positive IBC in our study. Additional improvements in HER2-positive survival could be attributed to use of trastuzumab in the relapsed or metastatic setting. Further survival improvements in HER2-positive IBC could be expected with use of trastuzumab at diagnosis, widely implemented in 2005. Validation of these findings from other population-based studies is needed, in order to help characterize the true prognostic and predictive factors for survival in IBC.

## Abbreviations

BC-SS: breast cancer-specific survival; CCR: California Cancer Registry; CI: confidence interval; EOD: extent of disease; ER: estrogen receptor; HER2: human epidermal growth factor-like receptor 2; IBC: inflammatory breast cancer; LABC: locally advanced breast cancer; MBC: metastatic breast cancer; NR: not reached; OS: overall survival; PR: progesterone receptor; SEER: Surveillance, Epidemiology, and End Results; SES: socioeconomic status; TNM: tumor node metastasis.

## Competing interests

The authors declare that they have no competing interests.

## Authors' contributions

JAZ developed the study design, and was involved with data collection, analysis, assembly and interpretation, manuscript drafting, and providing overall project support. WYT was involved with data analysis, assembly, and interpretation, and drafted the manuscript. THT collected, analyzed, and interpreted study data. RSM developed the study design, was involved in data assembly and interpretation, and drafted the manuscript. HA-C was involved with data collection and interpretation. All authors read and approved the final manuscript.
